# AGING AND POSITIVE EMOTIONS: WHAT’S LOVE GOT TO DO WITH IT?

**DOI:** 10.1093/geroni/igae098.1479

**Published:** 2024-12-31

**Authors:** Joseph Mikels

**Affiliations:** DePaul University, Chicago, Illinois, United States

## Abstract

Aging is associated with improvements in well-being; healthy older adults report a balance of fewer negative emotional experiences relative to positive emotional experiences. Additionally, meaningful social connections become prioritized as we grow older. Taken together, patterns in social and emotional aging would suggest that older adults might experience more love than their younger counterparts. But what is love? Recent theoretical and empirical advances conceptualize love as positivity resonance – a caring interpersonal connection involving shared positivity and synchrony. In three studies, the experience of positivity resonance in older adults was examined. In the first pilot study, 44 older adults who participated in a group exercise program reported experiencing positivity resonance 77.15% of the time during the program, which is higher than previously reported levels positivity resonance in a younger sample (65.93%). In a second survey study with 184 older adults (M age = 69.45) and 223 younger adults (M age = 24.74), older adults reported significantly higher trait-level perceived positivity resonance (M = 70.25) compared to younger adults (M = 53.39), t(405) = -9.11, p <.001. In a third study in which 100 older (M age = 74.67) and 100 younger participants (M age = 22.40) walked with a partner, older adults reported higher episode-level positivity resonance (M = 88.07) compared to younger adults (M = 83.41), t(195) = 2.00, p =.047. Across these three unique and methodological distinct studies, it appears that older adults do indeed experience more love than their younger counterparts.

